# Separate analysis of human papillomavirus E6 and E7 messenger RNAs to predict cervical neoplasia progression

**DOI:** 10.1371/journal.pone.0193061

**Published:** 2018-02-21

**Authors:** Shuling Liu, Takeo Minaguchi, Bouchra Lachkar, Shuang Zhang, Chenyang Xu, Yuri Tenjimbayashi, Ayumi Shikama, Nobutaka Tasaka, Azusa Akiyama, Manabu Sakurai, Sari Nakao, Hiroyuki Ochi, Mamiko Onuki, Koji Matsumoto, Hiroyuki Yoshikawa, Toyomi Satoh

**Affiliations:** 1 Doctoral Program in Obstetrics and Gynecology, Graduate School of Comprehensive Human Sciences, University of Tsukuba, Tsukuba, Ibaraki, Japan; 2 Department of Obstetrics and Gynecology, Faculty of Medicine, University of Tsukuba, Tsukuba, Ibaraki, Japan; 3 Department of Obstetrics and Gynecology, Showa University School of Medicine, Tokyo, Japan; 4 Ibaraki Prefectural Central Hospital, Kasama, Ibaraki, Japan; Fondazione IRCCS Istituto Nazionale dei Tumori, ITALY

## Abstract

A few studies previously suggested that human papillomavirus (HPV) E6 messenger RNA (mRNA) may exist uniformly in all grades of cervical intraepithelial neoplasia (CIN), whereas the detection rate of E7 mRNA may increase with disease progression from low-grade CIN to invasive carcinoma. The aim of this study was to clarify the different roles of E6 and E7 mRNAs in cervical carcinogenesis. The presence of each E6 and E7 mRNA was analyzed in 171 patients with pathologically-diagnosed CIN or cervical carcinoma. We utilized a RT-PCR assay based on consensus primers which could detect E6 mRNA (full-length E6/E7 transcript) and E7 mRNAs (spliced E6*/E7 transcripts) separately for various HPV types. E7 mRNAs were detected in 6% of CIN1, 12% of CIN2, 24% of CIN3, and 54% of cervical carcinoma. The presence of E7 mRNAs was significantly associated with progression from low-grade CIN to invasive carcinoma in contrast with E6 mRNA or high-risk HPV (HR-HPV) DNA (p = 0.00011, 0.80 and 0.54). The presence of both E6 and E7 mRNAs was significantly associated with HPV16/18 DNA but not with HR-HPV DNA (p = 0.0079 and 0.21), while the presence of E6 mRNA was significantly associated with HR-HPV DNA but not with HPV16/18 DNA (p = 0.036 and 0.089). The presence of both E6 and E7 mRNAs showed high specificity and low sensitivity (100% and 19%) for detecting CIN2+ by contrast with the positivity for HR-HPV DNA showing low specificity and high sensitivity (19% and 89%). The positive predictive value for detecting CIN2+ was even higher by the presence of both E6 and E7 mRNAs than by the positivity for HR-HPV DNA (100% vs. 91%). In 31 patients followed up for CIN1-2, the presence of both E6 and E7 mRNAs showed significant association with the occurrence of upgraded abnormal cytology in contrast with E6 mRNA, HR-HPV DNA, or HPV16/18 DNA (p = 0.034, 0.73, 0.53, and 0.72). Our findings support previous studies according to which E7 mRNA is more closely involved in cervical carcinogenesis than E6 mRNA. Moreover, the separate analysis of E6 and E7 mRNAs may be more useful than HR-HPV DNA test for detecting CIN2+ precisely and predicting disease progression. Further accumulation of evidence is warranted to validate our findings.

## Introduction

Cervical cancer is the fourth most commonly diagnosed cancer and the fourth most common cause of cancer death in women worldwide [1]. Each year, 528,000 women develop cervical cancer, and 266,000 women die of the disease, accounting for 7.5% of all cancer deaths in females [1]. Human papillomavirus (HPV) is classified by the sequence of the L1 gene. Infection with high-risk HPV (HR-HPV), including types 16 and 18, causes development of low-grade cervical intraepithelial neoplasia (CIN), and viral persistence induces cellular transformation resulting in progression to high-grade CIN and invasive cervical cancer [2]. HPV viral genome has 6 early genes, E1, E2, E4, E5, E6, and E7, and 2 late genes, L1 and L2, encoding capsid proteins. Among the early genes, E6 and E7 cause cancer by inactivating the tumor suppressor proteins p53 and Rb, respectively [3]. Normal epithelial cells persistently infected with HR-HPV first develop low-grade CIN. When viral DNA is integrated into host chromosome, constant overexpression of E6 and E7 induces abnormal proliferation, transformation and immortalization, and inhibits differentiation, apoptosis and immune response, leading to development of high-grade CIN. Accumulation of genetic and epigenetic alterations further causes progression to invasive cancer [4]. E6 is mainly expressed from full-length E6/E7 mRNA, and E7 is mainly expressed from spliced E6*/E7 mRNA [5]. HPV16 expresses two isoforms of E7 gene, and the other HPV types including HPV18 express one isoform of E7 gene. To date, only a few studies previously investigated the distinct roles of E6 and E7 mRNAs for cervical carcinogenesis [6, 7]. According to Nakagawa et al., E6 transcript is uniformly detected from CIN1 to invasive cancer, but E7 transcripts show a higher detection rate with disease progression from low-grade CIN to invasive cancer [6]. Another previous publication by Sotlar et al. showed that detection rate of E7 transcript increased with disease progression in contrast with E6 mRNA showing only moderate increase [7]. The aim of our study was to investigate the distinct roles of each E6 and E7 mRNAs in the pathogenesis of cervical cancer.

## Materials and methods

### Patients and samples

**Fig 1:**
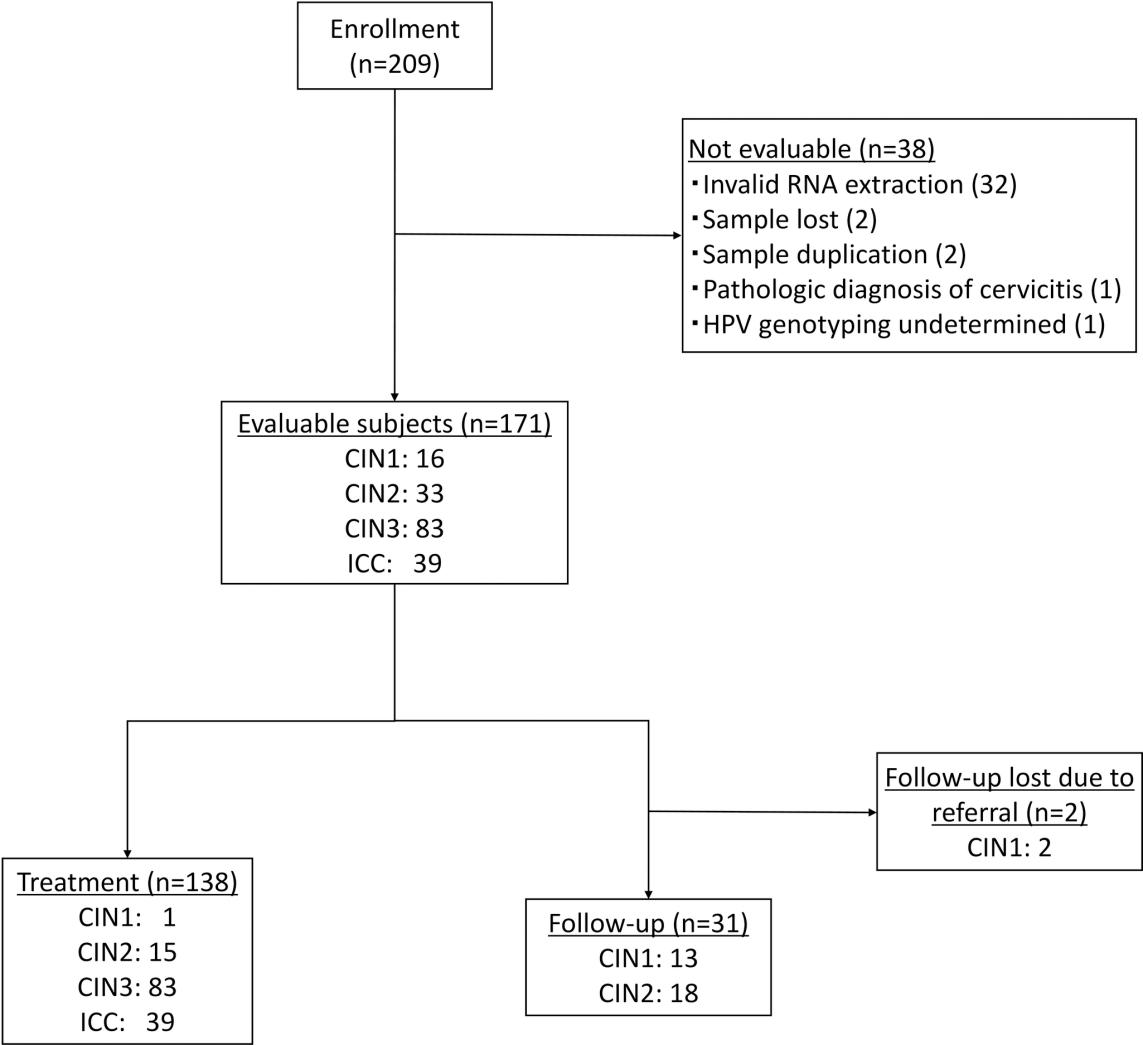
Study design. HPV: human papillomavirus, CIN: cervical intraepithelial neoplasia, ICC: invasive cervical cancer.

The current study comprised two parts: a cross-sectional study of analyzing E6/E7 mRNAs in cervical specimens from patients with CIN or invasive cervical carcinoma and an adjunctive longitudinal study of following up patients with CIN1-2 (Fig 1). Women with histologically and newly diagnosed CIN or cervical carcinoma were eligible to participate in this study and recruited between December 2014 and April 2017 at the outpatient clinic of University of Tsukuba Hospital. The study population was composed of CIN1 (n = 16), CIN2 (n = 33), CIN3 (n = 83) and cervical carcinoma (n = 39). The median age was 41.0 years for CIN1 (range 23–59), 33.0 years for CIN2 (range 22–65), 36.0 years for CIN3 (range 22–70), and 49.0 years for cervical carcinoma (range 33–76). Cervical specimens were collected with a Rovers Cervex-Brush (Rovers Medical Devices, Oss, The Netherlands) into a ThinPrep vial containing PreservCyt solution (HOLOGIC, Tokyo, Japan). Cells were immediately collected and stored in -80°C until use. Study protocol was approved by the Ethics Committee University of Tsukuba Hospital (H26-119). Written informed consent was obtained prior to enrollment of participants. Histology was evaluated based on the most severe lesion present. Cytology was classified according to the Bethesda system [8]. The included patients were treated or followed-up according to the clinical guidelines [9]. Study results of the mRNA analyses did not influenced their management. The median follow-up duration was 194 days (range 0–613). Follow-up data were retrieved until 2017-5-31.

### DNA extraction and HPV genotyping

Genomic DNA was extracted using SepaGene kit (Eidia, Tokyo, Japan) according to the manufacturer’s instruction. HPV genotyping was performed by L1-PCR and RFLP analyses as described previously [6] or at a clinical testing laboratory (SRL, Tokyo, Japan) by Amplicor linear array HPV genotyping test (Roche Diagnostics, Tokyo, Japan). HR-HPVs are defined as HPV 16, 18, 31, 33, 35, 39, 45, 51, 52, 56, 58, 59 and 68, which can be detected by Hybrid Capture 2 (HC2).

### RNA extraction and Reverse Transcriptase PCR (RT-PCR)

Total RNA extraction and DNase treatment were performed as described previously [6]. RT-PCR was conducted using OneStep RT-PCR kit (QIAGEN, Tokyo, Japan) according to the manufacturer’s instruction. We utilized a RT-PCR assay based on consensus primers designed to maintain around 80–90% homology to the known conserved sequences in E6 and E7 ORFs among multiple oncogenic HPVs [6]. E6 and E7 mRNAs could be separately detected for at least HPV types 16, 18, 31, 33, 35, 51, 52, 56, 58 and 59 [6]. We used β2-microglobulin as a control for RT-PCR in order to validate normal RNA extraction and no contamination of DNA which will affect the RT-PCR results, as E6/E7 DNA is the same size as E6 mRNA. Primers used for RT-PCR and PCR are as follows: E6/E7, ACC GAA AAC GGT TGA ACC GAA AAC GGT and GAG CTG TCG CTT AAT TGC TC; β2-microglobulin, TGT CTT TCA GCA AGG ACT GG and GAT GCT GCT TAC ATG TCT CG.

### Statistical analysis

Differences in proportions were evaluated by the Fisher’s exact test. Diagnostic indices of sensitivities, specificities, positive predictive values, and negative predictive values with 95% confidence intervals were calculated for detecting CIN2+, CIN3+, and invasive cervical cancer. Disease progression of CIN1-2 was examined as a surrogate by the Kaplan-Meier method calculating the intervals from E6/E7 sample collection until patients showed upgraded results of Pap test compared with the cytology at E6/E7 sample collection or they were censored, and the difference was statistically evaluated by the log-rank test.

## Results

**Fig 2:**
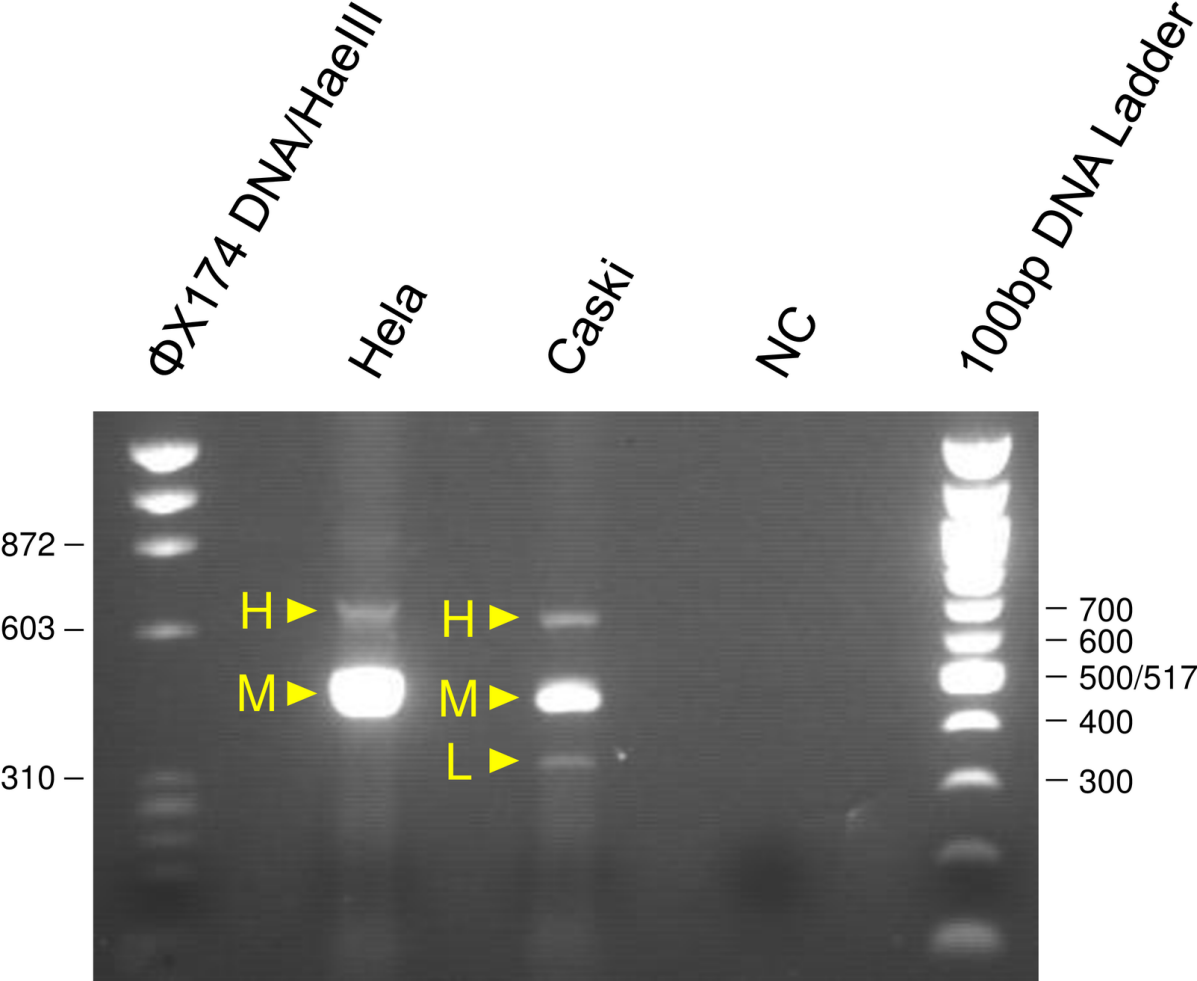
E6/E7 mRNA expression patterns by RT-PCR in human cervical cancer cell lines. H: full-length E6/E7 (E6), M: spliced E6*I/E7 (E7), L: spliced E6*II/E7 (E7).

**Fig 3:**
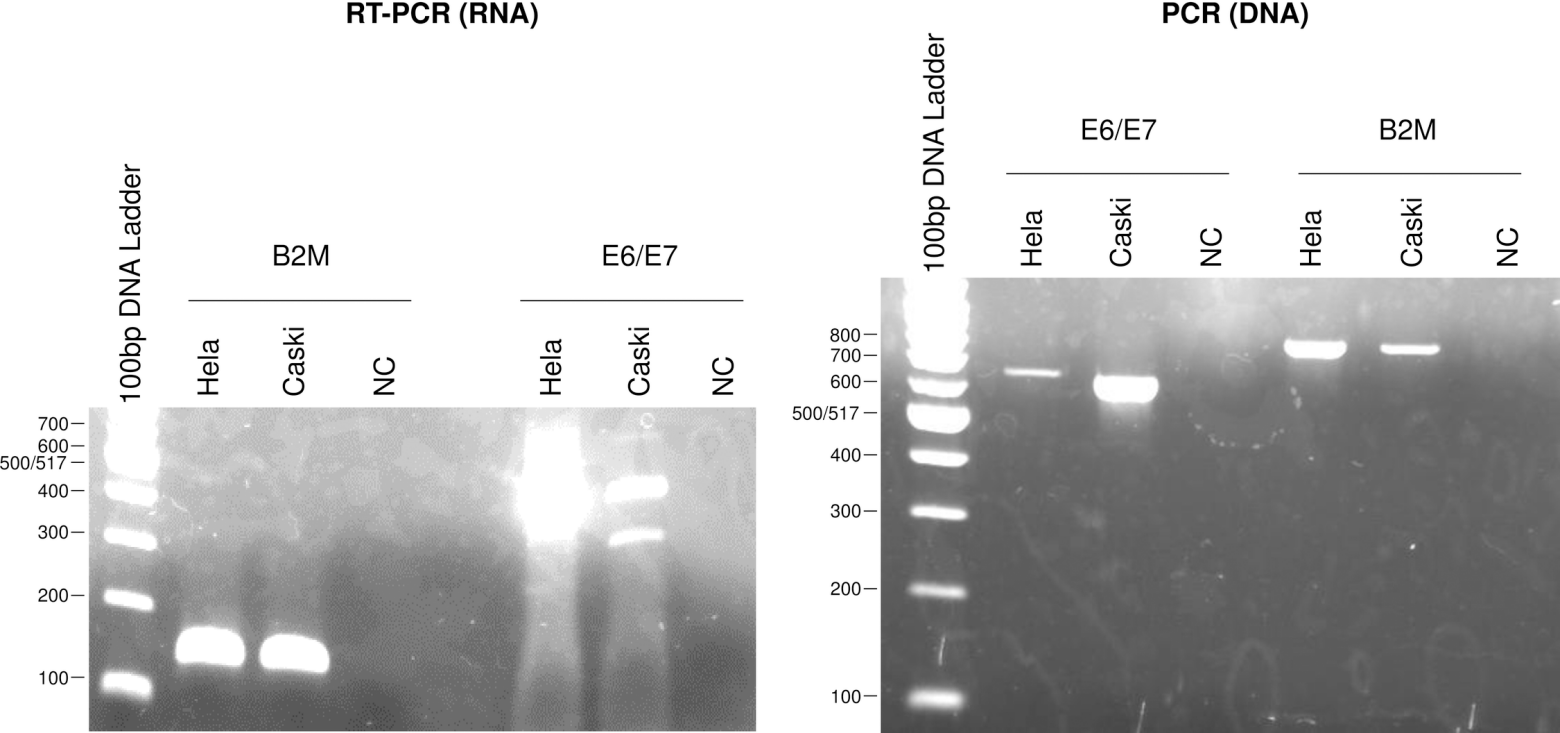
Comparison between DNA and RNA of HPV E6/E7 and human β2-microglobulin (B2M) genes. The size of E6/E7 DNA is 652bp for HeLa and 622bp for CaSki, same as E6 mRNA. The size of B2M DNA is 775bp and B2M RNA is 148bp.

We first analyzed the E6 and E7 mRNA expression patterns in human cervical cancer cell-lines CaSki and HeLa by RT-PCR (Fig 2), and confirmed that the expression patterns were consistent with data published by Nakagawa et al. [6]. In addition to E6 mRNA, two isoforms of E7 mRNA were detected in HPV 16-positive CaSki cells, and one isoform of E7 mRNA detected in HPV 18-positive HeLa cells. In order to verify that our RT-PCR assay works properly, we further performed sequencing analyses of E6/E7 cDNAs and confirmed that E6 mRNA is actually full-length E6/E7 transcript and that E7 mRNAs are actually spliced E6*/E7 transcripts. The E6/E7 DNA is the same size as the full-length RNA, 652 bp for HeLa and 622 bp for CaSki. β2-microglobulin is 148 bp for RNA and 775 bp for DNA (Fig 3).

**Fig 4:**
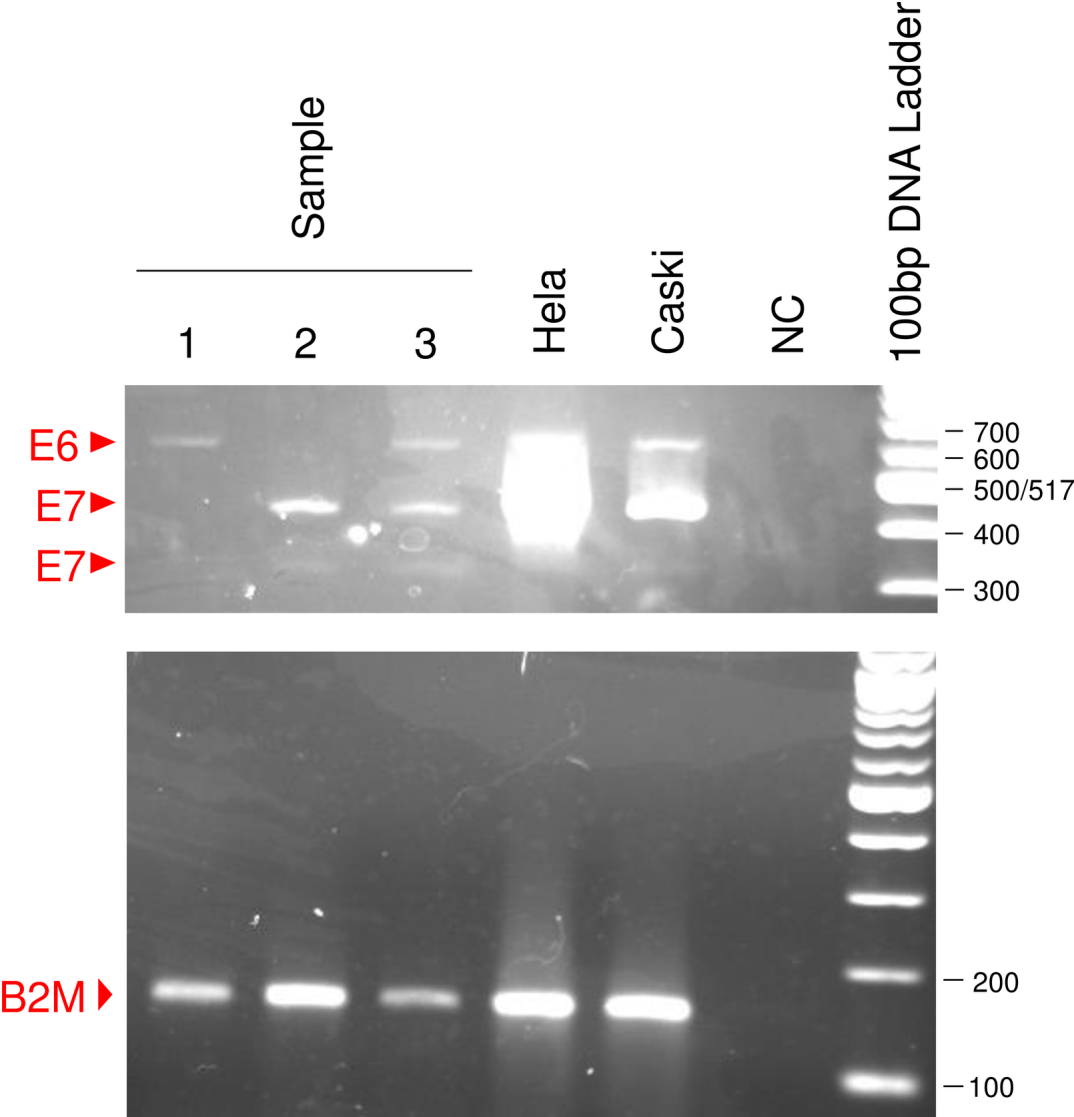
Detection of E6/E7 mRNAs from patients. E6 transcript is detected in samples 1 and 3, and 2 kinds of E7 transcript are detected in samples 2 and 3.

Subsequently we analyzed the E6/E7 mRNA expressions in liquid-based cytology samples from 171 patients. Fig 4 shows an example of detection of E6/E7 mRNA from patients. Beta2-microglobulin amplification showed no contamination by genomic DNA in every sample. The detection rate of E7 mRNA significantly increased with disease progression from low-grade CIN to invasive cancer, while those of E6 mRNA and HR-HPV DNA did not change (p = 0.00011, 0.80 and 0.54, respectively; Table 1). We next examined the relationship between E6/E7 mRNA expressions and HPV genotypes. The presence of E6 mRNA showed significant associations with the positivity for HR-HPV DNA but not with the positivity for HPV16/18 DNA (p = 0.0036 and 0.089; Table 2), whereas the presence of both E6 and E7 mRNAs showed significant associations with the positivity for HPV16/18 DNA but not with the positivity for HR-HPV DNA (p = 0.0079 and 0.21; Table 2).

**Table 1:** E6/E7 mRNA analyses and HPV genotyping in LBC samples from patients with cervical neoplastic diseases.

	CIN1	%	CIN2	%	CIN3	%	ICC	%	*P*-value
E6 mRNA(+)	7/16	44	16/33	48	35/83	42	20/39	51	0.80
E7 mRNA(+)	1/16	6	4/33	12	20/83	24	21/39	54	0.00011
E6 mRNA(+) and E7 mRNA(+)	0/16	0	4/33	12	12/83	14	14/39	36	0.0047
E6 mRNA(+) and/or E7 mRNA(+)	8/16	50	17/33	52	43/83	52	27/39	69	0.27
HR-HPV DNA(+)	13/16	81	30/33	91	75/83	90	33/39	85	0.54
HPV16/18 DNA(+)	2/16	13	12/33	36	36/83	43	25/39	64	0.030

Abbreviations: mRNA = messenger RNA; HPV = human papillomavirus; LBC = liquid-based cytology; CIN = cervical intraepithelial neoplasia; ICC = invasive cervical cancer; HR-HPV = high-risk HPV.

**Table 2:** Relationship between E6/E7 mRNAs and HPV genotypes.

	HR-HPV DNA			HPV16/18 DNA		
	(+)	(-)	*P*-value	(+)	(-)	*P*-value
E6 mRNA(+)	75/151 (50%)	3/20 (15%)	0.0036	40/75 (53%)	38/96 (40%)	0.089
E7 mRNA(+)	40/151 (26%)	6/20 (30%)	0.79	26/75 (35%)	20/96 (21%)	0.056
E6 mRNA(+) and E7 mRNA(+)	29/151 (19%)	1/20 (5%)	0.21	20/75 (27%)	10/96 (10%)	0.0079
E6 mRNA(+) and/or E7 mRNA(+)	86/151 (57%)	8/20 (40%)	0.16	46/75 (61%)	48/96 (50%)	0.16

Abbreviations: mRNA = messenger RNA; HPV = human papillomavirus; HR-HPV = high-risk HPV.

Next, we examined diagnostic accuracies for cervical neoplastic diseases by E6/E7 mRNA analyses. For detecting CIN2+, the presence of both E6 and E7 mRNAs showed high specificity and low sensitivity (100% [95% confidence interval [10], 79–100] and 19% [95% CI, 13–26]; Table 3) in contrast with the positivity for HR-HPV DNA showing high sensitivity and low specificity (19% [95% CI, 4–46] and 89% [95% CI, 83–93]; Table 3). Notably, the positive predictive value (PPV) for detecting CIN2+ was even higher by the presence of both E6 and E7 mRNAs than by the positivity for HR-HPV DNA or HPV16/18 DNA (100%, 91% and 91%, respectively; Table 3). Similar trends were also observed about the diagnostic accuracies for detecting CIN3+ and invasive cervical cancer (Tables 4 and 5).

**Table 3:** Diagnostic indices of E6/E7 mRNA analyses for detecting CIN2+.

	Sensitivity	Specificity	PPV	NPV
	(% [95% CI])	(% [95% CI])	(% [95% CI])	(% [95% CI])
E6 mRNA(+)	71/155 (46 [38–54])	9/16 (56 [30–80])	71/78 (91 [82–96])	9/93 (10 [5–18])
E7 mRNA(+)	45/155 (29 [22–37])	15/16 (94 [70–100])	45/46 (98 [88–100])	15/125 (12 [7–19])
E6 mRNA(+) and E7 mRNA(+)	30/155 (19 [13–26])	16/16 (100 [79–100])	30/30 (100 [88–100])	16/141 (11 [7–18])
E6 mRNA(+) and/or E7 mRNA(+)	86/155 (55 [47–63])	8/16 (50 [25–75])	86/94 (91 [84–96])	8/77 (10 [5–19])
HR-HPV DNA(+)	138/155 (89 [83–93])	3/16 (19 [4–46])	138/151 (91 [86–95])	3/20 (15 [3–38])
HPV16/18 DNA(+)	73/155 (47 [39–55])	14/16 (88 [62–98])	73/75 (97 [91–100])	14/96 (15 [8–23])

Abbreviations: mRNA = messenger RNA; CIN = cervical intraepithelial neoplasia; PPV = positive predictive value; NPV = negative predictive value; CI = confidence interval; HR-HPV = high-risk HPV.

**Table 4:** Diagnostic indices of E6/E7 mRNA analyses for detecting CIN3+.

	Sensitivity	Specificity	PPV	NPV
	(% [95% CI])	(% [95% CI])	(% [95% CI])	(% [95% CI])
E6 mRNA(+)	55/122 (45 [36–54])	26/49 (53 [38–67])	55/78 (71 [59–80])	26/93 (28 [19–38])
E7 mRNA(+)	41/122 (34 [25–43])	44/49 (90 [78–97])	41/46 (89 [76–96])	44/125 (35 [27–44])
E6 mRNA(+) and E7 mRNA(+)	26/122 (21 [14–30])	45/49 (92 [80–98])	26/30 (87 [69–96])	45/141 (32 [24–40])
E6 mRNA(+) and/or E7 mRNA(+)	70/122 (57 [48–66])	25/49 (51 [36–66])	70/94 (74 [64–83])	25/77 (32 [22–44])
HR-HPV DNA(+)	108/122 (89 [81–94])	6/49 (12 [5–25])	108/151 (72 [64–79])	6/20 (30 [12–54])
HPV16/18 DNA(+)	61/122 (50 [41–59])	35/49 (71 [57–83])	61/75 (81 [71–89])	35/96 (36 [27–47])

Abbreviations: mRNA = messenger RNA; CIN = cervical intraepithelial neoplasia; PPV = positive predictive value; NPV = negative predictive value; CI = confidence interval; HR-HPV = high-risk HPV.

**Table 5:** Diagnostic indices of E6/E7 mRNA analyses for detecting invasive cervical cancer.

	Sensitivity	Specificity	PPV	NPV
	(% [95% CI])	(% [95% CI])	(% [95% CI])	(% [95% CI])
E6 mRNA(+)	20/39 (51 [35–68])	74/132 (56 [47–65])	20/78 (26 [16–37])	74/93 (80 [70–87])
E7 mRNA(+)	21/39 (54 [37–70])	107/132 (81 [73–87])	21/46 (46 [31–61])	107/125 (86 [78–91])
E6 mRNA(+) and E7 mRNA(+)	14/39 (36 [21–53])	116/132 (88 [81–93])	14/30 (47 [28–66])	116/141 (82 [75–88])
E6 mRNA(+) and/or E7 mRNA(+)	27/39 (69 [52–83])	65/132 (49 [40–58])	27/94 (29 [20–39])	65/77 (84 [74–92])
High-risk HPV DNA(+)	33/39 (85 [69–94])	14/132 (11 [6–17])	33/151 (22 [16–29])	14/20 (70 [46–88])
HPV16/18 DNA(+)	25/39 (64 [47–79])	82/132 (62 [53–70])	25/75 (33 [23–45])	82/96 (85 [77–92])

Abbreviations: mRNA = messenger RNA; PPV = positive predictive value; NPV = negative predictive value; CI = confidence interval; HR-HPV = high-risk HPV.

**Fig 5:**
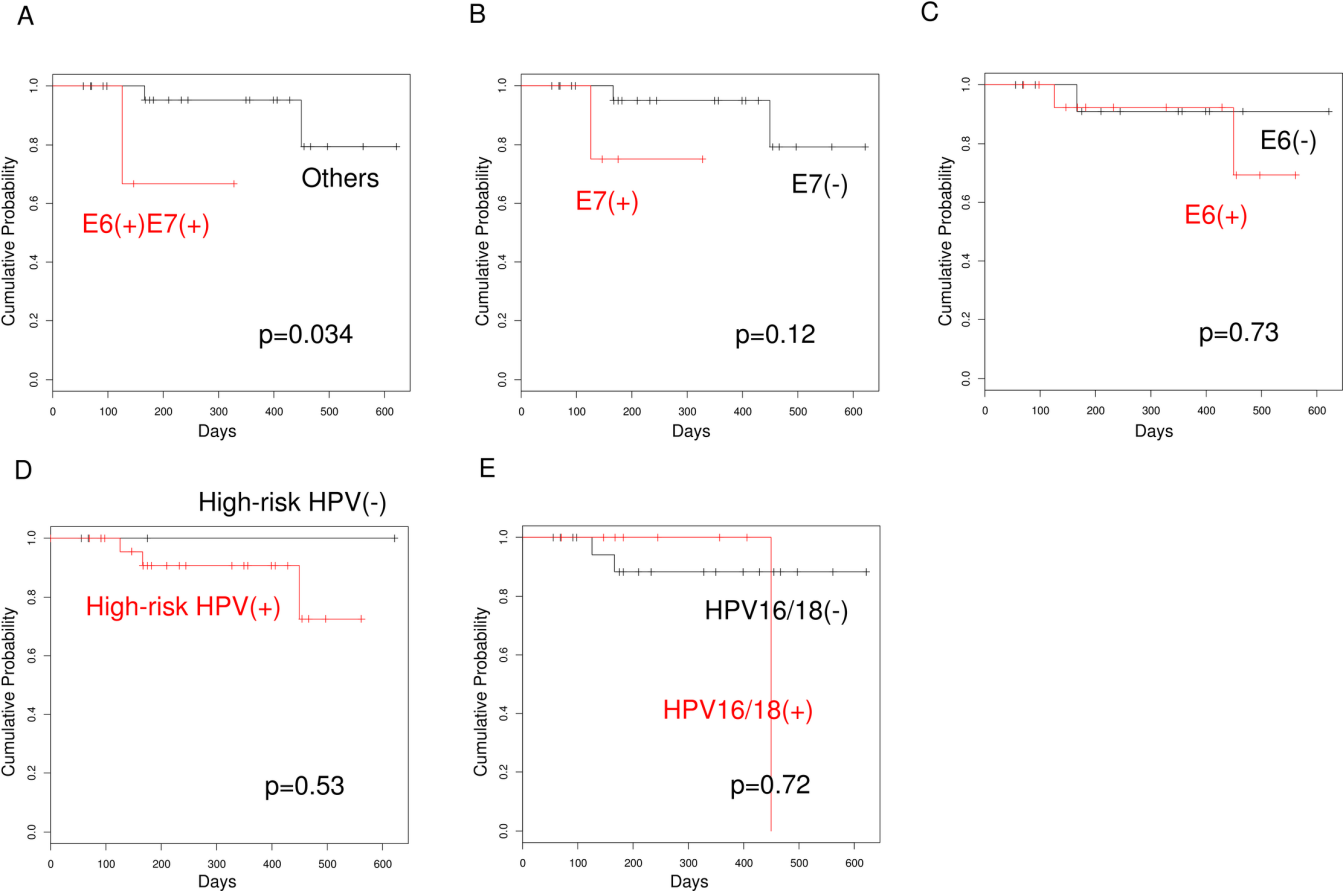
Kaplan-Meier curves for upgraded Pap-test results in followed-up patients with CIN1-2. *A*, cases positive for both E6 and E7 mRNAs (n = 3) *vs*. the remainder (n = 28); *B*, cases with positive E7 mRNAs (n = 4) *vs*. negative E7 mRNAs (n = 27); *C*, cases with positive E6 mRNA (n = 15) *vs*. negative E6 mRNA (n = 16); *D*, cases with positive HR-HPV DNA (n = 26) *vs*. negative HR-HPV DNA (n = 5); *E*, cases with positive HPV16/18 DNA (n = 8) vs. negative HPV16/18 DNA (n = 23).

Finally, we examined the impact of the positivity for E6/E7 mRNAs or specific HPV genotypes on disease progression by following up 31 patients with CIN1-2. Since no disease progression was pathologically diagnosed yet in those patients, we compared intervals until the occurrence of upgraded abnormal cytology compared with the cytology at E6/E7 sample collection as a surrogate for disease progression. The presence of both E6 and E7 mRNAs showed significant association with the occurrence of upgraded abnormal cytology, the presence of E7 mRNAs showed association without statistical significance, but the presence of E6 mRNA, HR-HPV DNA, or HPV 16/18 DNA showed no such trends (p = 0.034, 0.12, 0.73, 0.53, and 0.72, respectively; Fig 5).

## Discussion

Our E6/E7 RT-PCR analyses showed that E7 mRNAs were significantly associated with progression from low-grade CIN to invasive carcinoma in contrast with E6 mRNA showing no such trend (Table 1). Furthermore, we found that the presence of E6 mRNA was significantly associated with the positivity for HR-HPV DNA but not with the positivity for HPV16/18 DNA, whereas the presence of both E6 and E7 mRNAs was significantly associated with the positivity for HPV16/18 DNA but not with the positivity for HR-HPV DNA (Table 2). These observations suggest that E7 mRNA may be more closely involved in cervical carcinogenesis than E6 mRNA and that the presence of both E6 and E7 mRNAs may be the oncogenic property specific for HPV16/18, keeping in line with previous publications where the expression of E7 by itself can immortalize human keratinocytes at a low frequency but E6 has no such activity, and the combination of E6 and E7 is highly efficient at immortalizing most types of primary cells [11, 12]. Additionally in the transgenic mouse model, E7 alone, but not E6 alone, is reported to be sufficient to induce high-grade CIN and invasive cervical cancers and the addition of E6 results in larger and more extensive cervical cancers [13]. Oncoproteins E6 and E7 are known to cause development of cervical cancer by inactivating the tumor suppressors p53 and Rb, respectively. Accordingly, our findings suggest that Rb may play a more critical role in cervical carcinogenesis than p53, being consistent with the published finding that Rb and Ki67 were the strongest predictive markers for CIN progression among various molecular markers including p53 [14].

Diagnostic indices by our E6/E7 RT-PCR analyses for detecting cervical neoplastic diseases exhibited that the presence of both E6 and E7 mRNAs had high specificity and low sensitivity in contrast with the positivity for HR-HPV DNA having high sensitivity and low specificity (Tables 3–5). HC2 is indeed reported to show high sensitivity and relatively low specificity (88.8–95.8% and 38.7–56% for CIN2+) [15–18]. Notably, the PPV for detecting CIN2+ by the presence of both E6 and E7 mRNAs was even higher than by the positivity for HR-HPV DNA (Table 3). Accordingly, the separate analysis of E6 and E7 mRNAs may be more useful than HR-HPV test for detecting CIN2+ precisely. As with the presence of both E6 and E7 mRNAs, liquid-based cytology test is also reported to have high specificity for detecting cervical neoplastic diseases (84.8–94.1% for CIN2+) [19]. However, while cytology test is considered to reflect the present status of diseases, E6/E7 mRNA analysis may be able to predict future disease progression, as this test examines HPV oncogene expressions with transforming abilities. In this context, we further examined the impact of the presence of E6/E7 mRNAs on disease progression by following up patients with CIN1-2. The presence of both E6 and E7 mRNAs showed significant associations with the occurrence of upgraded abnormal cytology, the presence of E7 mRNA showed association without statistical significance, while positive E6 mRNA, HR-HPV DNA, or HPV 16/18 DNA showed no such trends (Fig 4). Regarding follow-up study of HPV mRNA tests, the longitudinal studies have reported that positive mRNA at baseline is an excellent predictor for future development of CIN2+ or CIN3+ in referral or post-treatment populations [20–26]. Moreover, a recent longitudinal screening study has reported that the Aptima HPV test, which collectively detects E6/E7 mRNAs from 14 types of HR-HPV, has a similar sensitivity for detection of CIN2+ or CIN3+ and a significantly higher specificity than the HC2 test [27]. Together with these published findings, our above observations suggest that the separate analysis of E6 and E7 mRNAs may predict disease progression of CIN more precisely than HPV DNA tests. However, further following up patients and pathologically detecting disease progression are required to clarify the predictive significance of separately analyzing E6 and E7 mRNAs.

The sensitivity of our E6/E7 mRNA test for detecting CIN2+ is lower than those of other reported HPV RNA tests (77.0–96.3% for CIN2+) [10, 15–18, 23]. However, while almost all other HPV RNA tests examine E6 and E7 mRNAs collectively, our RT-PCR system can detect each E6 and E7 mRNAs separately so that disease progression may be more precisely predicted by individually evaluating E7 mRNA which appears more closely involved in cervical carcinogenesis than E6 mRNA. Moreover, our system using liquid-based cytology specimens will be suitable for clinical application by a “one sample for all” approach.

In conclusion, our separate analyses of E6/E7 mRNAs demonstrated here that the presence of E7 mRNAs was significantly associated with progression from low-grade CIN to invasive carcinoma in contrast with positive E6 mRNA or HR-HPV DNA. Besides, the presence of both E6 and E7 mRNAs was significantly associated with the positivity for HPV16/18 DNA, while the presence of E6 mRNA was significantly associated with the positivity for HR-HPV DNA. The presence of both E6 and E7 mRNAs showed high specificity and low sensitivity for detecting CIN2+ by contrast with the positivity for HR-HPV DNA. Furthermore, the presence of both E6 and E7 mRNAs showed significant association with the occurrence of upgraded abnormal cytology in the patients followed-up for CIN1-2 by contrast with positive E6 mRNA, HR-HPV DNA, or HPV16/18 DNA. Our findings suggest a closer involvement of E7 mRNAs than E6 mRNA in cervical carcinogenesis. Moreover, the separate analysis of E6 and E7 mRNAs may be a more useful tool than HR-HPV DNA test for detecting CIN2+ precisely and predicting disease progression. Further accumulation of evidence is warranted to validate our proposal.
